# A Study of Polish Family with Scoliosis and Limb Contractures Expands the MYH3 Disease Spectrum

**DOI:** 10.3390/genes15010125

**Published:** 2024-01-19

**Authors:** Justyna Frasuńska, Agnieszka Pollak, Paweł Turczyn, Anna Kutkowska-Kaźmierczak, Jakub Pepłowski, Rafał Płoski, Beata Tarnacka

**Affiliations:** 1 Department of Rehabilitation, Medical University of Warsaw, 02-091 Warsaw, Poland; frasunska@gmail.com (J.F.); beata.tarnacka@wum.edu.pl (B.T.); 2 Department of Medical Genetics, Medical University of Warsaw, 02-091 Warsaw, Poland; rploski@wp.pl; 3 Clinic of Early Arthritis, National Institute of Geriatrics, Rheumatology and Rehabilitation, 02-637 Warsaw, Poland; pawel.turczyn@spartanska.pl; 4 Department of Medical Genetics, Institute of Mother and Child, 01-211 Warsaw, Poland; anna.kutkowska@imid.med.pl; 5 The Rare Diseases Laboratory, Laboratory of Genetics, University Center for Laboratory Medicine, University Clinical Centre of the Medical University of Warsaw, 02-097 Warsaw, Poland; jakub.peplowski@uckwum.pl

**Keywords:** *MYH3* pathogenic variant, next-generation sequencing, congenital scoliosis, congenital contractures

## Abstract

A disease associated with malfunction of the MYH3 gene is characterised by scoliosis, contractures of the V fingers, knees and elbows, dysplasia of the calf muscles, foot deformity and limb length asymmetry. The aim of this study was to identify the cause of musculoskeletal deformities in a three-generation Polish family by exome sequencing. The segregation of the newly described c.866A>C variant of the MYH3 gene in the family indicates an autosomal dominant model of inheritance. The detected MYH3 variant segregates the disease within the family. The presented results expand the MYH3 disease spectrum and emphasize the clinical diagnostic challenge in syndromes harbouring congenital spine defects and joint contractures.

## 1. Introduction

The causes underlying congenital spine defects are heterogeneous and comprise a variety of environmental and genetic factors. Environmental factors have been proven to have a strong influence on the development of congenital scoliosis. Among the most important are hypoxia, vitamin A deficiency, alcohol use, smoking, valproic acid, boric acid and hyperthermia. The rank of environmental factors is significantly lower for confirmed genetic disease with spinal defects as a symptom [1]. Furthermore, spine defects may be a component of various disease entities such as hemivertebra, Klippel–Feil syndrome, Chiari malformation, tethered cord, spina bifida, diastematomyelia and many others [2]. Congenital curvature of the spine in the form of scoliosis occurs in 11.4% of all cases of congenital spine defects [2]. Some of these deformities are caused by genetic syndromes related to pathogenic variants in many genes, i.e., the fibrilin-1 gene (*FBN1*) in 5q23, the fibriline–2 gene (*FBN2*) in 5q23 or the myosin heavy chain (*MYH3)* in 17p13. 

The *MYH3* gene encodes the protein known as the embryonic myosin heavy chain. The role of MYH3 in the development of the skeletal and muscular systems is of significant importance. This protein is composed of various components, including a globular head, a neck domain, a hinge and a long coiled-coil rod domain. The head contains an actin filament binding site and is crucial for ATP hydrolysis, which is essential for muscle contraction. Within the neck, there are IQ motifs that have the ability to bind to calmodulin. The coiled-coil rod plays a mechanical role in the formation of stiff fibre bundles [3,4]. During normal human development, expression is restricted to stages of embryonic development and rapidly declines after birth [5,6]. In humans, MYH3 expression predominates in myotubes fated to become fast myofibers, subsequently replaced by the expression of different myosins (MYH1, MYH2 and MYH4). While one or more of these myosins may contribute to the development of structurally normal skeletal muscle in individuals with *MYH3* pathogenic variants, the function of foetal muscles rich in fast-twitch myofibers is functionally impaired [3]. 

Congenital diseases associated with the *FBN1*, *FBN2* and *MYH3* genes include, respectively, Marfan syndrome, Beals syndrome and distal arthrogryposis type 8. These disorders share many similar defects, such as scoliosis, joint limpness, joint contractures, arachnodactyly and muscle hypoplasia, often making their accurate diagnosis difficult [2]. Many of these defects can be diagnosed prenatally. Ultrasonography plays an important role in this process. The precise detection of the cause of a defect is possible by applying comprehensive genetic testing such as whole-exome sequencing (WES). In many cases, comprehensive genetic tests are not performed, and the molecular cause of a deformity is not established; thus, the disorder causing the deformity is classified as idiopathic. The difficulty of identifying and preventing these diseases is also related to their sporadic as well as familial occurrence. Despite significant improvement in prenatal testing, there is still no fully effective treatment, and unfortunately, severe disability and impaired quality of life for affected children are common. 

## 2. Materials and Methods

The following study was conducted in cooperation with the Department of Medical Genetics, Medical University of Warsaw. A total of 11 family members of the proband were recruited for genetic testing. Informed consent for the presentation of clinical and medical data was obtained from all subjects involved in the study.

### 2.1. The Proband

A 41-year-old male was admitted to our Department of Rehabilitation, Medical University of Warsaw, for the diagnosis and treatment of severe scoliosis and intermittent significant spinal pain. During the examination, a number of dysmorphic features besides scoliosis were noted, which suggested the presence of a genetic syndrome. 

### 2.2. The Proband’s Family

After interviewing the patient, it was determined that other family members also presented clinical abnormalities (the proband’s father and three of the proband’s siblings); thus, they underwent a detailed clinical evaluation. The presence of scoliosis, camptodactyly and other deviations was confirmed. All the aforementioned family members were referred for genetic testing. Three of the proband’s children, who do not present characteristic phenotypic features, also underwent additional genetic testing. Initially, no genetic testing was performed on the children of the remaining siblings of the proband. Recently, genetic testing was performed on three children of the proband’s sisters (III.4, III.5, III.7) who have similar clinical symptoms. 

### 2.3. Genetic Testing

**Figure 1:**
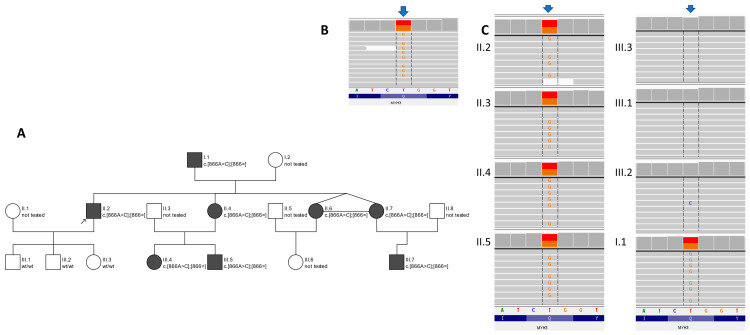
Family pedigree accompanied by a graphical presentation of the c.866A>C variant in *MYH3* gene created in Integrative Genomics Viewer v.2.9.4. Family pedigree—(**A**) panel. Circles represent females and squares represent males. The filled symbols show affected individuals. Proband is marked with a black arrow. Results of WES in proband—(**B**) panel, results of amplicon deep sequencing in proband and relatives—(**C**) panel. Members of the family are marked in accordance with the numbering on the pedigree. Arrows indicates position of the c.866A>C variant.

DNA from the proband and his family (Figure 1A) was obtained from peripheral blood and extracted with a standard protocol. Library preparation for WES was performed on the proband’s DNA sample with SeqCap EZ MedExome probes (Roche, Basel, Switzerland) targeting the human exome with enhanced coverage for clinically relevant genes. Subsequently, paired-end index sequencing (2 × 100) was performed on the Illumina sequencer (Illumina Inc., San Diego, CA, USA) to obtain 85,896,495 reads (89.5% of the target bases were covered at a minimum of 20×, whereas 97.5% had a coverage of minimum 10×). The raw data were processed with the pipeline described previously [7]. Variants were inspected with an Integrative Genomics Viewer (IGV). The heterozygous variants within the *MYH3* gene (NM_002470.4:c.866A>C, p.Gln289Pro) were prioritised for further verification by amplicon deep sequencing (ADS) in the proband and his relatives (i.e., all kids, siblings and father) (Figure 1B). ADS confirmed the presence of the *MYH3* variant in the proband’s DNA and revealed that it is also present in affected all of his family members (namely, his father and siblings) (Figure 1A,C). The population frequency for the variant c.866A>C was 0 in gnomAD (database, v.3, https://gnomad.broadinstitute.org/, accessed on 19 November 2023) and 0.0001 in the in-house datasets of >4500 WES of Polish individuals. To date, the c.866A>C variant within *MYH3* has not been described in The Human Gene Mutation Database Professional v.2023.1. All applied bioinformatics algorithms marked it as pathogenic/damaging/disease-causing (the Combined Annotation Dependent Depletion (CADD) score was 26.6; the Rare Exome Variant Ensemble Learner (REVEL) score was 0.923). According to ACMG criteria [8], the c.866A>C variant in the *MYH3* gene is finally rated as likely pathogenic (total score of 7 points, class 4 in accordance with IARC recommendations [9]). 

## 3. Results

### 3.1. Clinical Features

Upon physical examination, three siblings (two of them were monozygotic twins) and the subject’s father were found to have similar symptoms. The detailed clinical characteristics of the affected family members are presented in Table 1. Based on the symptoms and skeletal deformities in the twins, Beals syndrome was initially diagnosed in early childhood, which was revised after the WES results in the adult patients. The clinical diagnostic score for Beals syndrome was 10 points for all the adult family members [10].

### 3.2. Clinical Characteristics of Patients

The presence of scoliosis, camptodactyly, joint contractures, dysplasia of the calf muscles, asymmetry of the limb lengths, short stature, short neck and dysmorphic changes in the head (downslating palpebral fissures) were found. The clinical features of the five patients are show in Table 1. No intellectual disability was found, and all the adults in the family have tertiary education. 

All the family members suffered from scoliosis, contractures of several joints, camptodactyly, drooping outer corners of the eyes, low posture and a short neck. 

The greatest severity of defects was observed in the twins studied (subjects II.6 and II.7). Both sisters had advanced scoliosis (operated on at 15 and 16 years of age), divergent strabismus (operated on at 5 years of age) and one of the twins also had surgery for a cleft soft palate (at 2 years of age). The third sibling sister also had strabismus surgery at the age of 5 years.

In all the family members, we observed right-convex thoracic scoliosis, whose secondary lumbar scoliosis, convex to the left, was compensated. The greatest degree for scoliosis was presented by the twins (the Cobb angle was above 40 degrees [11]). Both sisters had surgical treatment of scoliosis: subject II.7 at 16 years of age, posterior stabilisation of the spine between Th2 and L3, and subject II.6 at 15 years, posterior stabilisation of the Th3 and L2 spinal nerves. In the other family members, the severity of scoliosis was less and did not require surgical treatment. 

The skeletal changes differed slightly between the different family members. Imaging studies showed spinal defects located primarily in the upper cervical and lower lumbar regions, and they mainly involved the location of the occipitovertebral and lumbosacral transitions. These spinal defects included:

- Bony blocks or partial bony blocks between the occipitocervical junctions; the C1 vertebra and C2 vertebra; the L4 vertebra; the L5 vertebra; and the lumcosacral junctions (all subjects);
- Unconjoined/hypoplasia of the posterior arch of the C1 vertebra (subjects II.4 and II.7);
- Spina bifida of the L5 and S1 vertebra (subject II.4);
- Narrow vertebra canal (subjects I.1. and II.2);
- Lumbar vertebrae with atypical morphology (all subjects);
- Congenital lower hypoplastic intervertebral discs (all subjects).

**Figure 2:**
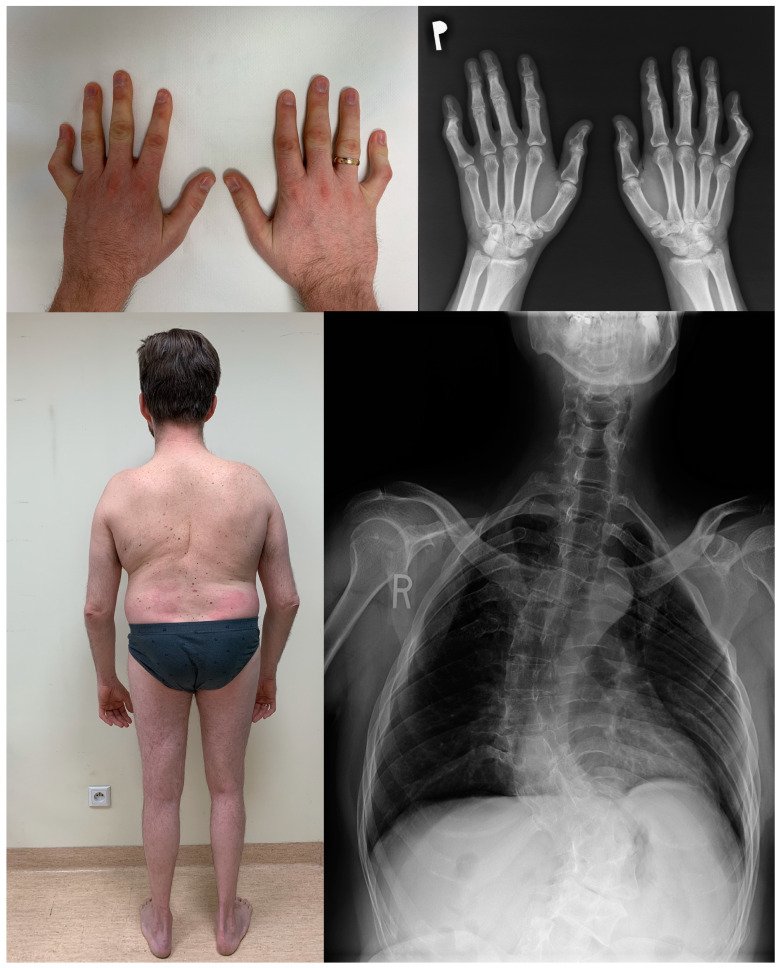
Phenotypic and radiological features of a family harbouring a pathogenic variant in the *MYH3* gene.

Images of phenotypic and radiographic features of the family are presented in Figure 2.

All the patients studied had contractures in the shoulder, elbow, interphalangeal, hip and ankle joints. A lack of full extension at the elbows, hips and knees included values below 20 degrees of joint mobility. Camptodactyly in four of the family members was most advanced in the V fingers, and in one person (subject II.7), in the II-IV fingers. In two of the family members harbouring the *MYH3* variant, three additional isolated foot defects were observed (Table 1).

Subject II.6 (a woman) presented the most severe symptoms. She was born with the lowest weight and height compared to the other siblings. Furthermore, she was the only one of her siblings to be diagnosed with other medical problems. She had a cleft palate operated on at two years old and was burdened with severe bronchial asthma. Additionally, in childhood, she had multiple stays in pulmonology clinics. Due to asthma and deformation of the chest as a result of severe scoliosis, the patient still has a breathing disorder. She was not diagnosed with any cardiac defects or other severe heart diseases. 

In subject II.6, small mitral and tricuspid regurgitations with a history of sinus tachycardia were described in childhood with an echo-controlled heart. Also, subject II.4 has small mitral, aortic and tricuspid regurgitations.

The children’s father (subject I.1) has additionally been diagnosed with asthma, diabetes, glaucoma, hypertension and myasthenia gravis. Subjects II.2 and II.6 were also diagnosed with hypertension. Some of the family members (subjects II.4 and II.7) also have autoimmune thyroiditis. 

The children of patient II.2 were developing correctly. Patients III.4 (13 years), III.5 (6 years) and III.7 (2 years) had similar clinical symptoms. Patient III.6 had no clinical symptoms, and therefore the *MYH3* gene was not tested in her.

## 4. Discussion

The genetic testing of eleven members of a three-generation family detected a causative variant within the *MYH3* gene in eight individuals (Figure 1). Here, we present the first description of the c.866A>C variant within the *MYH3* gene. To date, four disease syndromes caused by pathogenic variants in the *MYH3* gene have been described (MIM*160720). Table 2 shows the differential phenotypic features of other pathogenic variants of this gene [4,12,13,14,15,16], including the one in our study.

During a study conducted on our family in the 1980s, an initial diagnosis of Beals syndrome was proposed based on the characteristic clinical features of Beals syndrome observed in subject II.2 [17], which have never been reported in the literature so far in patients harbouring *MYH3* pathogenic variants (Table 2). Thus, the presented phenotype expands the phenotypic spectrum of *MYH3*-related disorders. It should be emphasised that the clinical presentations in patients harbouring pathogenic variants within the *MYH3* gene vary from a complex syndrome (i.e., Freeman–Sheldon or Sheldon–Hall) to early-onset scoliosis [18]; every novel distinctive finding in *MYH3* is important for more precise and faster diagnosis. 

The novel c.866A>C *MYH3* missense variant described in this study is located within the motor domain of the embryonic myosin heavy chain, adjacent to the previously described c.875C>G and c.859T>G variants. Both variants are established causes of Arthrogryposis distal type 2B [3,19] and Sheldon–Hall syndrome with vertebral fusions [20], respectively. The phenotypes have many overlapping features (short stature, multiple contractures, etc.), but elongation of the phalanges and abnormal pigmentation of the skin have never been reported to date. 

## 5. Conclusions

Our case supports and expands the unusual phenotypic variability in *MYH3*-related disorders. Further studies are required to understand this phenomenon.

## Figures and Tables

**Table 1:** Clinical (phenotypic and radiological) features of presented patients with pathogenic variant within the *MYH3* gene.

		Subject	I.1	II.2	II.4	II.6	II.7	III.4	III.5	III.7
Biological attributes		sex	Male	Male	Female	Female	Female	Female	Male	Female
		age (years)	69	41	38	31	31	13	6	2
Stature and trunk	Physical finding	short stature	+	+	+	+	+	−25c	25c	75c
		short neck	+	−	+	+	+	+	+	−
	Radiological finding	scoliosis	+	+	+	+	+	−	mild	mild
		vertebral fusion	+	+	+	−	+	+	−	?
Face	Physical finding	downslating palpebral fissures	+	+	+	+	+	+	+	+
Upper limb	Physical finding	joint contractures	+	+	+	+	+	−	+	+
		camptodactyly	+	+	+	+	+	+	−	−
		elongation of the proximal and middle phalanges of the hands	−	+	−	−	−	−	−	−
	Radiological finding	carpal fusion	+	−	−	+	−	?	?	?
Lower limb	Physical finding	dysplasia of the calf muscles	+	+	+	+	+	+	+	−
		joint contractures	+	+	+	+	+	+	+	mild
		hallux vagus	+	−	+	−	−	+	−	−
		partial syndactyly of the II and III toes	−	+	−	−	−	−	−	−
		Elongation of the proximal phalanges and metatarsal bones	−	+	−	−	−	−	+	−
	Radiological finding	peripheral instability with plantar deviation of the toes at the IP I joints with predominance of the right foot	−	−	+	−	−	+	−	−
		tarsal fusion	−	−	−	−	−	?	?	?

+ feature present, − feature absent, ?-unknown, c-centile.

**Table 2:** Differential phenotypes of *MYH3*-gene-related diseases; abbreviations: n.d.—no data.

Phenotype	Arthrogryposis, Distal, Type 2A (Freeman–Sheldon) [4]	Arthrogryposis, Distal, Type 2B3 (Sheldon–Hall) [5,6]	Contractures, Pterygia and Spondylocarpotarsal Fusion Syndrome 1A [7,8,9]	Contractures, Pterygia and Spondylocarpotarsal Fusion Syndrome 1B [9]	Family Described within the Presented Study
inheritance	AD	AD	AD	AR	AD
face	very small mouth, pinched lips, H-shaped dimpling of the chin	Triangular face Facial contractures that result in deep nasolabial folds Attached earlobes Downslating palpebral fissures Broad bridge of nose Small mouth	Microcephaly (in some patients) Ptosis cleft palate Downslating palpebral fissures Low-set posteriorly rotted ears Hearing loss (in some patients)	Dysmorphic features Cleft palate (rare)	Downslating palpebral fissures
neck	Short neck	n.d.	Short neck Webbed neck	Short neck Webbed neck	Short neck
contractures	Multiple (shoulders, elbows, thumbs, hips, knees, toes)	Multiple (shoulders, elbows, fingers, hips, knees, feet)	Elbows, knees, hips (in same patients)	Variable (neck, shoulders, elbows, fingers, hips and/or knees)	Multiple (elbows, hands, fingers, hips, knees, feet)
spine	Kyphoscoliosis (frequently develops) Spina bifida occulta	Scoliosis (rare)	Scoliosis Vertebral fusion Hemivertebrae Spondylolisthesis (rare)	Scoliosis Vertebral fusion	Scoliosis
hands	Cortical thumbs Camptodactyly Ulnar deviation	Camptodactyly Ulnar deviation Palmar position of the thumb Overlapping finger	Carpal fusion Camptodactyly V finger clinodactyly	Carpal fusion	Camptodactyly and isolated defects, e.g., elongation of the phalanges
feet	Talipes equinovarus Contracted toes	Hallux vagus Talipes equinovarus	Tarsal fusion	Tarsal fusion Club foot (rare)	Isolated defects, e.g., hallux vagus, partial syndactyly, peripheral instability with plantar deviation of the toes
skin	Skin thickening on fingers	Hypoplastic or absent flexion creases of palms	Variable pterygia (neck, elbows and/or fingers)	Variable pterygia (neck, elbows, fingers and/ or knees)	Abnormal pigmentation Skin dryness
hight	Short stature	Short stature	Short stature	n.d.	Short stature

## Data Availability

Raw data are available upon request to the researchers. The data are not publicly available due to privacy restrictions.
